# Multiple scan averaging to yield accurate quantitative analysis of optical coherence tomography angiograms

**DOI:** 10.1038/s41598-020-62956-2

**Published:** 2020-04-10

**Authors:** Hafi M. Khan, Alex Gentle, James A. Armitage, Chi-ho To, Andrew K. C. Lam

**Affiliations:** 1 School of Medicine, Faculty of Health, Deakin University, Deakin, Australia; 2 School of Optometry, The Hong Kong Polytechnic University, Hong Kong, China

**Keywords:** Diseases, Medical research

## Abstract

Optical coherence tomography angiography (OCTA) is widely used in ophthalmic practice. Most OCTA studies based their findings on a single OCTA measurement. We conducted an observational study of 82 eyes from 82 healthy subjects to compare variations of OCTA parameters among five successive measurements. A 3 × 3 mm Early Treatment of Diabetic Retinopathy Study grid centred at fovea was used. An average from five successive OCTA measurements (both perfusion density and vessel density) was calculated to be used as the reference standard. There was no significant difference in perfusion and vessel densities among five successive OCTA measurements, and from different levels of averaging. Perfusion density was close to the reference standard when average from three measurements was used (discrepancy within 1.5%) as compared with using just one measurement (discrepancy from 3.2% to 4.5%). Vessel density was also close to reference standard when average from three measurements was used (within 0.8 mm^−1^) as compared with using just one measurement (2 mm^−1^). Software feature that allows OCTA devices to average quantitative parameters for analysis will be useful.

## Introduction

The use of optical coherence tomography angiography (OCTA) is becoming routine in ophthalmic practice. The rapid increase in use can be attributed to its non-invasive and fast acquisition properties, which allow for more frequent assessment of the ophthalmic vasculature without the concerns about allergic responses that are inherent to conventional fluorescein angiography^1^. Its main application is in the detection and monitoring of ocular and systemic diseases impacting the posterior segment^2^. However, OCTA may also prove useful for imaging the vasculature in diseases that impact the anterior segment^3^.

In ophthalmic practice, it is common that a reported measurement refers to the average result of several successive measurements. For example, in applanation tonometry, practitioners usually take two to three intraocular pressure (IOP) measurements to generate an average result to the IOP for a patient^4,5^; average results from three to five measurements are commonly required in clinical protocols for the use of ocular biometers^6^; three successive bidirectional applanation measurements are recommended in determining corneal hysteresis^7,8^ and in pulsatile ocular blood flow assessment, an average of three successive measurements are necessary to detect a difference between normal subjects and glaucoma patients^9^.

Current OCTA technology still has considerable scope for further enhancement, for example through reducing eye movement and projection artefacts, and segmentation errors during acquisitions^2^. Furthermore, Lei, *et al*.^10^ found that vessel density was influenced by the signal strength of the image, however there is currently no standard signal strength requirement across different OCTA devices. It is therefore interesting that, unlike the other ophthalmic assessments described above, almost all OCTA studies based their findings on a single OCTA measurement^11–13^, and it must be assumed that the collection of only one OCTA measurement remains the norm in clinical practice. Lei, et al^10^. proposed that repeatability (defined as the agreement between same successive measurements of a subject performed by the same operator and device under the same conditions), reproducibility (defined as the agreement between the same measurements of a subject when performed by a different operator or using a different device) and discrepancy (the extent of variability when multiple readings of the same measurement are averaged) are important reliability factors to consider for accurate analysis of quantitative parameters.

Recently, studies have reported the advantages of combining several OCTA frames to generate an averaged image for analysis^14–16^. The image quality was found to be better from an averaged image, and vessel metrics were significantly different when compared with those from a single frame. However, combining OCTA frames requires post hoc analysis using a separate imaging analysis tool such as ImageJ, which effectively precludes its use in a clinical setting. There are also different threshold strategies and binarisation settings which create difficulties when comparing across different studies. Nowadays, most of the OCTA machines come with their built-in software containing OCTA metrics. Such advancement provides detailed measurements of retinal structures and vascular morphology for clinical decision-making. However, there is no agreement between clinicians or researchers on the minimum number of scans needed to reliably attain a “true value” of the measurement of interest. It is therefore important to understand the benefit of averaging several OCTA measurements over taking one single OCTA measurement and averaging several OCTA measurements. It may be easier for practitioners to simply average OCTA measurements from several scans in order to reduce any discrepancy. The purpose of this study was therefore to determine the minimum number of successful OCTA measurements necessary for acceptable accuracy during OCTA assessment.

## Methods

Healthy subjects aged 18–50 years were included in the study. Exclusion criteria included major ocular and retinal pathology (such as, but not limited to, retinal scars, cataract, and glaucoma), history of previous ocular surgery, presence of vascular disease (conditions such as diabetes, ocular vascular occlusions), premature birth (prior to 36 weeks gestation), and history of myopia control interventions. There was no restriction on the degree of refractive error. One hundred and seven subjects, of whom 96 subjects fulfilled the inclusion criteria, were recruited for the study, through Deakin University, Australia and The Hong Kong Polytechnic University. Although both eyes were measured, only data from 82 of the right eyes were included in the analysis (explained below). Motion tracking and fixation were controlled for by the built-in machine software (Cirrus 5000 with AngioPlex, Zeiss, Jena). No mydriatic agent was used during the image acquisition. The study was conducted according to the tenets of the Declaration of Helsinki, and received approval from Departmental Research Committee of School of Optometry, The Hong Kong Polytechnic University, and Institutional Review Board of Deakin University. All subjects gave informed consent prior to participation in the study.

### Image acquisition

The OCTA imaging sequence consisted of five 3 × 3 mm angiography B-scans centred at the fovea. The foveal centre was identified by the device software during and after the image acquisition which employed structural OCT to identify the foveal region. No manual adjustment of the foveal location was carried out. These scans were successive and performed by the same trained operator and device model for all the subjects at each participating centre. The testing was conducted in a dark room and compensated for subject’s spherical equivalent refractive error. Scans with signal strength less than 8/10 were rejected, which resulted in data from only 82 of 96 eyes being included.

**Figure 1:**
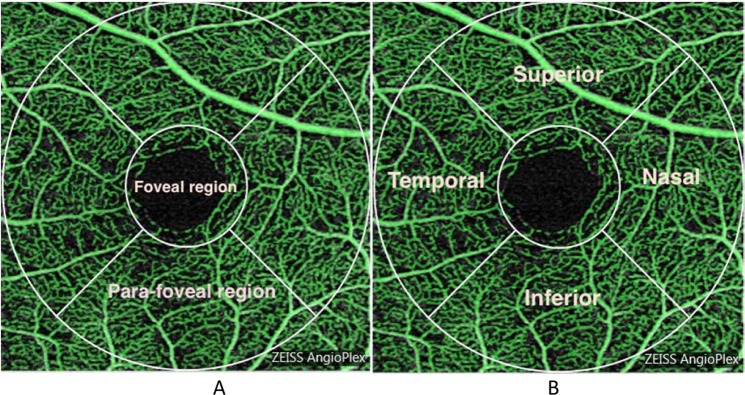
(**A**,**B**) Pictorial representation of a 3 × 3 mm square grid of the macular vasculature (right eye) as measured with OCTA. The vessels in a 3 mm diameter circle centred at the foveal region were analysed. The central foveal region depicted has a nominal diameter of 1 mm (**A**). The annulus, subdivided into four regions, has a nominal width of 1 mm. The para-foveal region covers the entire annulus of the four regions (**B**).

All the quantitative metrics were derived from the instrument software. The Cirrus OCTA software has the capability to provide Perfusion density (total area of perfused vasculature per unit area in the region of measurement) and Vessel density (total length of perfused vasculature per unit area in the region of measurement). These measurements are provided for various macula tissue subfields based on the Early Treatment of Diabetic Retinopathy Study (ETDRS) grid. The present study analysed the differences between the five scans in the 3 mm circle (aggregated foveal and para-foveal region, Fig. 1), the 1 mm to 3 mm annulus (para-foveal region), the central 1 mm circle (foveal region) and also the four regions surrounding the foveal region (Fig. 1). The vasculature beyond the 3 mm circle was not analysed.

Numerical OCTA data were exported as an XML file for each subject to be read into the manufacturer produced reader for analysis. All relevant patient codes, scan information, and numerical analysis of angiography parameters were exported to this reader. The vasculature analysed by the instrument’s software included data for the superficial capillary plexus, which was anatomically defined as part of the inner retina including the internal limiting membrane to inner plexiform layer layers. The layer boundary was automatically detected by the software. The vessels in the outer retina or the deeper layers were not analysed due to instrument limitations. Given the minimal magnification effects of axial length on vessel density in 3 × 3 mm scans magnification corrections were not applied^17^.

### Data analysis

The Kolmogorov-Smirnov test was used to determine distribution of the data. Repeated measures analysis of variances or Friedman tests were used to study difference among five individual measurements when data distribution was normal or not normal, respectively. Test-retest reliability of five successive measurements was studied using intraclass correlation coefficient (ICC) which was calculated from two-way random-effects model using a consistency definition. Coefficient of variation (CoV) was also calculated as standard deviation of five successive measurements divided by their average. Averaged results were generated with a variable number of measurements (first two, three, four, and all five) used for averaging to compare the effect of different levels of averaging. An average from five successive OCTA measurements (both perfusion density and vessel density) was calculated to be used as the reference standard. Parametric or non-parametric tests were used to study difference among five levels of averaging (first measurement and four averaged results). Assuming that averaging five measurements would provide the most accurate OCTA metrics (reference standard), agreement between OCTA metrics from averaging five measurements under the various averaging conditions was examined using Bland-Altman plots. To investigate the extent of discrepancy with averages of different numbers of measurements, the mean of the difference ±1.96 × standard deviation of the difference (95% limits of agreement) was calculated.

## Results

Results were presented in three different ways. Firstly, OCTA results from individual measurements were tabulated and graphically presented, followed by results from different levels of averaging. Finally, comparison between the reference standard and different levels of averaging was shown. The five successive OCTA measurements were similar without any significant difference at the aggregated, para-foveal, and foveal regions (Table 1). Table 1 shows ICC and CoV of aggregated, para-foveal and foveal regions for all 82 eyes. The ICC was all greater than 0.85. The CoV ranged from 3.1% to 7.7% between different regions of the ETDRS 3 mm grid for perfusion density. This was comparable to the CoV for vessel density. The CoV at the foveal region was greater than the aggregated and para-foveal regions for both perfusion density and vessel density, while the CoV for the para-foveal and aggregated regions was similar.

**Table 1:** Mean, ± standard deviation, perfusion density (%) and vessel density (mm^−1^) of five successive measurements at the ETDRS 3 mm grid from 82 eyes.

	Perfusion density (%)		
	Aggregated region	Para-foveal region	Foveal region
1^st^ measurement	37.6 ± 2.5	39.6 ± 2.4	21.3 ± 5.5
2^nd^ measurement	37.6 ± 2.0	39.7 ± 1.9	21.2 ± 5.2
3^rd^ measurement	37.5 ± 2.3	39.6 ± 2.3	21.2 ± 5.1
4^th^ measurement	37.6 ± 2.3	39.7 ± 2.2	21.4 ± 5.3
5^th^ measurement	37.7 ± 2.2	39.8 ± 2.1	21.2 ± 5.5
ICC	0.888	0.879	0.971
CoV	3.1%	2.9%	7.7%
Analysis	p = 0.474	p = 0.591	p = 0.978*
	**Vessel density (mm**^**−1**^**)**		
	**Aggregated region**	**Para-foveal**	**Foveal**
1^st^ measurement	21.3 ± 1.4	22.4 ± 1.4	12.5 ± 3.1
2^nd^ measurement	21.3 ± 1.1	22.4 ± 1.1	12.4 ± 2.9
3^rd^ measurement	21.3 ± 1.3	22.4 ± 1.3	12.4 ± 2.9
4^th^ measurement	21.2 ± 1.3	22.4 ± 1.3	12.5 ± 3.0
5^th^ measurement	21.3 ± 1.2	22.4 ± 1.2	12.4 ± 3.1
ICC	0.867	0.859	0.973
CoV	3.1%	3.0%	7.0%
Analysis	p = 0.466	p = 0.525	p = 0.994*

ICC: intraclass correlation coefficient.

CoV: coefficient of variation.

Analysis: Friedman’s tests; *repeated measures analysis of variance.

Within the para-foveal region sectors, as shown in Table 2, the superior region had the widest variations in both the perfusion density and vessel density among 5 measurements. Nevertheless, ICC and CoV were good. There were no significant differences yielded in all analyses.

**Table 2:** Mean ± standard deviation, perfusion density (%) and vessel density (mm^−1^) of five successive measurements at the para-foveal grid from 82 eyes.

	Perfusion density (%)			
	Superior	Inferior	Nasal	Temporal
1^st^ measurement	39.5 ± 3.0	39.5 ± 2.4	39.5 ± 2.5	40.1 ± 2.7
2^nd^ measurement	39.8 ± 2.1	39.5 ± 2.3	39.2 ± 2.8	40.1 ± 2.5
3^rd^ measurement	39.4 ± 2.7	39.4 ± 2.8	39.3 ± 3.0	40.3 ± 2.5
4^th^ measurement	39.4 ± 2.6	39.6 ± 2.7	39.2 ± 3.0	40.4 ± 2.3
5^th^ measurement	39.9 ± 2.5	39.7 ± 2.4	39.1 ± 3.7	40.4 ± 3.1
ICC	0.802	0.850	0.885	0.723
CoV	4.0%	3.7%	3.9%	4.0%
Analysis	p = 0.142	p = 0.529	p = 0.936	p = 523
	**Vessel density (mm**^**−1**^**)**			
	**Superior**	**Inferior**	**Nasal**	**Temporal**
1^st^ measurement	22.2 ± 1.7	22.3 ± 1.5	22.3 ± 1.4	22.8 ± 1.5
2^nd^ measurement	22.3 ± 1.3	22.3 ± 1.3	22.1 ± 1.5	22.8 ± 1.3
3^rd^ measurement	22.2 ± 1.6	22.3 ± 1.7	22.2 ± 1.5	22.9 ± 1.4
4^th^ measurement	22.1 ± 1.5	22.3 ± 1.5	22.2 ± 1.6	22.9 ± 1.3
5^th^ measurement	22.4 ± 1.3	22.4 ± 1.4	22.1 ± 1.9	22.8 ± 1.6
ICC	0.801	0.865	0.881	0.737
CoV	4.0%	3.7%	3.6%	3.8%
Analysis	p = 0.273	p = 0.921	p = 0.853	p = 0.894

ICC: intraclass correlation coefficient.

CoV: coefficient of variation.

Analysis: Friedman’s tests.

**Figure 2:**
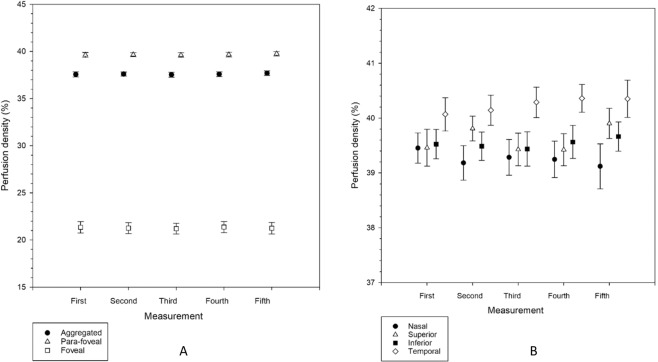
(**A**,**B**) Perfusion density from each of the five measurements in the aggregated, para-foveal, and foveal regions of a 3 × 3 mm ETDRS grid (**A**), and across individual sectors of the para-foveal region of a 3 × 3 mm ETDRS grid (**B**). Mean results are shown. Error bars indicate standard errors of the mean. There was no significant difference among the five measurements from repeated measures analysis of variance or Friedman tests (all p > 0.05).

**Figure 3:**
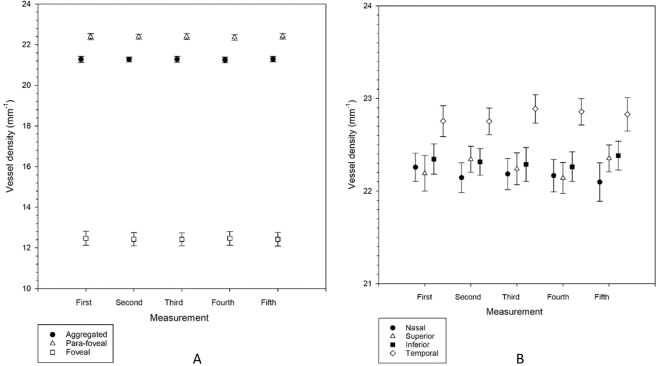
(**A**,**B**) Vessel density from each of the five measurements in the aggregated, para-foveal, and foveal regions of a 3 × 3 mm ETDRS grid (**A**), and across individual sectors of the para-foveal region of a 3 × 3 mm ETDRS grid (**B**). Mean results are shown. Error bars indicate standard errors of the mean. There was no significant difference among the five measurements from repeated measures analysis of variance or Friedman tests (all p > 0.05).

Figures 2 and 3 show the perfusion density and vessel density, respectively, at the aggregated, para-foveal, and foveal region, as well as the four regions within the annulus. There was no significant difference among the five successive OCTA measurements (Friedman tests, p = 0.142 to 0.936).

**Figure 4:**
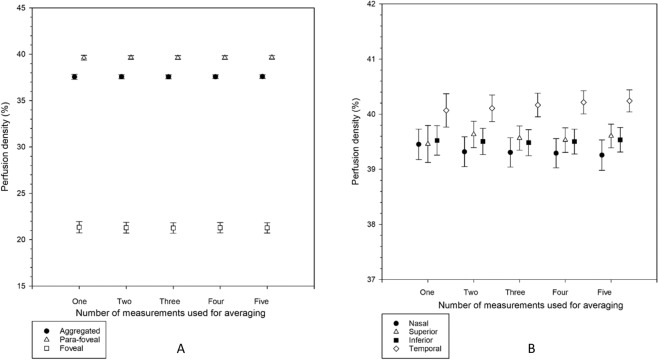
(**A**,**B**) Perfusion density from five levels of averaging (first measurement, average of the first two, first three, first four, and all five measurements in the aggregated, para-foveal, and foveal regions of a 3 × 3 mm ETDRS grid (**A**), and across individual sectors of the para-foveal region of a 3 × 3 mm ETDRS grid (**B**). Mean results are shown. Error bars indicate standard errors of the mean. There was no significant difference among the five levels of averaging from repeated measures analysis of variance or Friedman tests (all p > 0.05).

**Figure 5:**
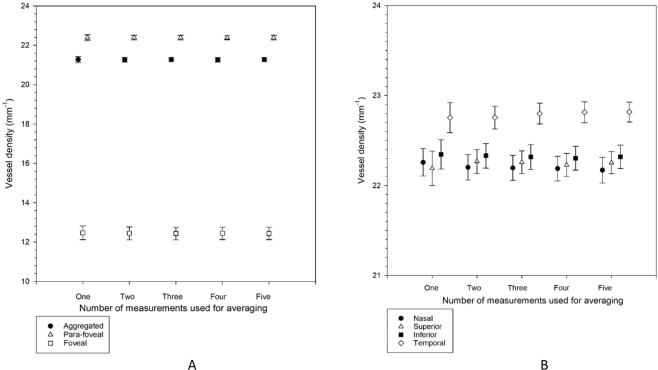
(**A**,**B**) Vessel density from five levels of averaging (first measurement, average of the first two, first three, first four, and all five measurements) in the aggregated, para-foveal, and foveal regions of a 3 × 3 mm ETDRS grid (**A**), and across individual sectors of the para-foveal region of a 3 × 3 mm ETDRS grid (**B**). Mean results are shown. Error bars indicate standard errors of the mean. There was no significant difference among the five levels of averaging from repeated measures analysis of variance or Friedman tests (all p > 0.05).

Figures 4 and 5 show results from different levels of averaging (first measurement, average of the first two, first three, first four, and all five measurements) in perfusion density and vessel density, respectively. There was no significant difference among different levels of averaging (p = 0.225 to 0.996).

**Figure 6:**
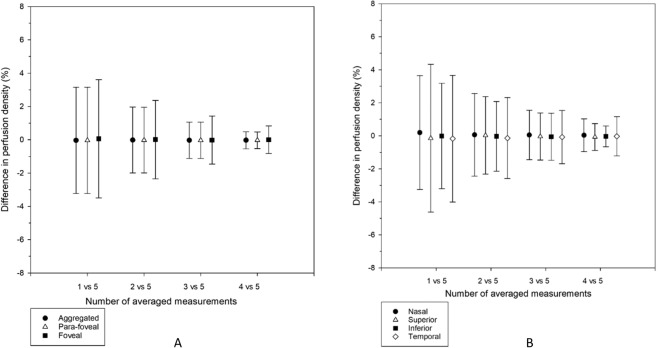
(**A**,**B**) Assuming that averaging five measurements would provide the most accurate perfusion density. Bland-Altman plots of agreement between averaged results from five measurements and different averaging conditions in the aggregated, para-foveal, and foveal regions of a 3 × 3 mm ETDRS grid (**A**), and across individual sectors of the para-foveal region of a 3 × 3 mm ETDRS grid (**B**). Mean differences are shown. Error bars indicate 95% limits of agreement.

**Figure 7:**
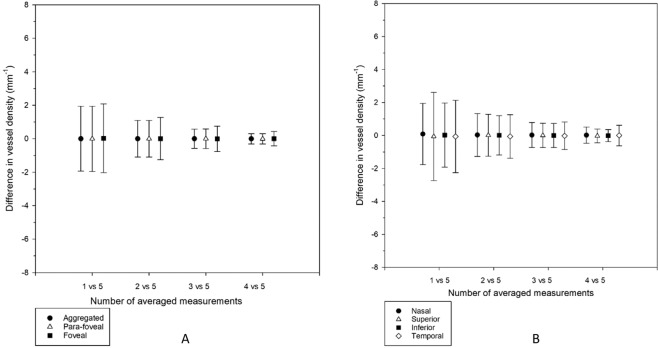
(**A**,**B**) Assuming that averaging five measurements would provide the most accurate vessel density. Bland-Altman plots of agreement between averaged results from five measurements and different averaging conditions in the aggregated, para-foveal, and foveal regions of a 3 × 3 mm ETDRS grid (**A**), and across individual sectors of the para-foveal region of a 3 × 3 mm ETDRS grid (**B**). Mean differences are shown. Error bars indicate 95% limits of agreement.

Compared with the reference standard (average from five successive measurements), the discrepancy was the greatest with the first measurement and the smallest with average of four measurements (Fig. 6 for Perfusion density, Fig. 7 for Vessel density). The reduction in discrepancy appeared uniformly across the four individual regions of perfusion and vessel densities, as well as across the whole ETDRS grid. In perfusion density, discrepancy with the first measurement was the highest in the superior region (4.5%) and the smallest in the inferior region (3.2%) (Fig. 6). The discrepancy with average of the first three measurements was similar among the four regions. Similarly, discrepancy in vessel density with the first measurement varied considerably among the four regions (Fig. 7). The greatest discrepancy was in the superior region (2.7 mm^−1^) and the smallest in the nasal region (1.9 mm^−1^). Discrepancy with the average of the first three measurements was similar among the four regions. Both perfusion and vessel densities had greater discrepancy at the foveal region compared with the aggregated and para-foveal regions. This could be attributed to the contribution of the foveal avascular zone (FAZ).

## Discussion

Variation of the intraocular pressure pulse under normal physiology may not allow one single tonometry measurement to accurately represent its true value. For this, or similar, reasons, some ophthalmic procedures require several successive measurements to be made to generate an average result. In the current study, no significant variation in average perfusion density and vessel density for all subjects across five successive OCTA measurements was found (Table 1, Figs. 2 and 3). This could be related to the magnitude of the OCTA parameters. Although Yu and Lam^9^ found significant reduction in IOP from successive measurements, repeated measurement of pulsatile ocular blood flow did not demonstrate any significant variation. Lack of significant difference could be due to different magnitudes of IOP (in an order of 13 mmHg) and pulsatile ocular blood flow (in an order of 730 µL min^−1^). Perfusion density and vessel density are in the order of 40% and 22 mm^−1^, respectively, such that greater variation would be needed to reveal a significant difference (Figs. 2 and 3).

We then treated an average of five successive measurements as the reference standard to represent the true or accurate value. Although there was no significant difference among different levels of averaging (Figs. 4 and 5), it was found that taking just one measurement could result in considerable deviation from the reference standard at different regions (Figs. 6 and 7). For example, practitioners would not be advised to take just one measurement for the superior region in studying perfusion and vessel densities. An important consideration in clinical practice is at what point a degree of statistical difference becomes clinically significant. Despite the ubiquity of OCT and OCTA in ophthalmic practice, no standardised protocol for scan acquisition or review exists^18^. Wang *et al*.^16^ reported that superficial vessel density acquired using their OCTA machine (similar to perfusion density in our study), in emmetropic and moderately myopic eyes along an elliptical annulus around fovea (similar to para-foveal region in our study), were 26.6% and 29.0%, respectively. When one perfusion density was determined at the para-foveal region, it could deviate from its true value by 3.2% (Fig. 6). Thus, taking only one measurement may render the technique unable to differentiate between normal vessel density differences between emmetropes and moderate myopes, such that the clinician cannot differentiate between physiological and pathological change. If an average from three measurements was used, the discrepancy was reduced to only 1.1%. Wang et al^19^. took a single OCTA measurement which could account for the absence of significant difference in perfusion density between the two groups.

Uji, *et al*.^15^ compared image quality and vessel metrics between one OCTA frame and average of up to 9 OCTA frames using a customised analysis routine. Vessel metrics in the superficial layer did not show further improvement after averaging 5 frames. However, the deep layer required averaging of up to 6 frames, presumably due to the lower fieldity at which this plexus can be resolved, due to lower signal strength induced by light attenuation and projection artefacts. Averaged images could also provide better visualization of the choriocapillaris^14^. It is a dilemma for practitioners whether to image a smaller field (e.g. 3 × 3 mm) at higher resolution (pixel/mm) or a bigger field (6 × 6 mm) at lower resolution. Uji, *et al*.^16^ found that averaging 3 OCTA frames of 6 × 6 mm could allow small capillaries discerned and the image quality was comparable to a single OCTA frame of 3 × 3 mm. Averaging just 3 OCTA frames could provide significant improvement in contrast-to-noise ratio. However, the challenge for practitioners is the necessary extra step of using separate image analysis tool such as ImageJ. Jung, *et al*.^1^ found that even though OCTA metric were significantly associated with best-corrected visual acuities in eye with retinal vein occlusion, averaging did not improve the association further when compared with a single-scan analysis.

To reduce discrepancy in OCTA measurements and attain a ‘true value’, many approaches can be taken such as capturing at higher-resolution to reduce speckle noise. However, this is not controlled by practitioners and usually embedded in instrument software. In a clinical setting, a simple way is to take several successive scans. Multiple scan averaging has demonstrated improved qualitative analysis of OCTA results^20,21^. Sakamoto, *et al*.^20^ showed that images successfully depicted the structures if produced by averaging multiple scans of identical retinal locations that could not be identified clearly in a single scan. Sander, *et al*.^21^ also showed that averaging several scans could improve image of retinal structures. By the same token and with advancement in OCTA technology capable of providing quantitative measurements, multiple scan averaging is a useful and simple approach. The underlying assumption being that the angiogram measurements are stochastically varying over the results taken. Therefore, in deciding whether to take two, three, four, or five scans, we propose that an average of three measurements is a reliable and effective ‘compromise’. From Figs. 6 and 7, an average of three measurements could reduce the discrepancy to half the magnitude of using one single scan (i.e. improved to within ±1.5% for perfusion density or ±0.8 mm^−1^ for vessel density). Dryness of the ocular surface from taking too many scans is another challenge to practitioners. Taking an average of four measurements did not improve the discrepancy too much especially for the four para-foveal sectors (Figs. 6B and 7B). Taking three measurements is practicable in real-life clinical practice where time-constraints, patient compliance and ‘chair fatigue’ are important factors to consider. Ideally, the machine software is able to readily compute an average from multiple scans.

Coscas, *et al*.^11^ studied the effect of aging on vessel density. They also reported their findings using a 3 × 3 mm ETDRS grid. Difference in vessel density in their study (similar to perfusion density in our study) between elderly (>60 years) and younger subjects varied from 3.3% (inferior) to 5.6% (superior). In the current study, a single measurement of perfusion density in the superior region was found to deviate from the reference value by as much as 4.5% (Fig. 6). Taking an average from three measurements resulted in the discrepancy being reduced to 1.4%. Similarly, the discrepancy reduced from 3.2% with one measurement to 1.4% with three measurements in the inferior region. Thus, it may not be possible to reveal an aging effect if only one measurement is taken as this single measurement may not represent the true value. This observation also applies to vessel density (Fig. 7).

The AngioVue (Optovue) and the AngioPlex (Zeiss Medical Technology) are the first two OCTA systems to be approved by the US Food and Drug Administration (FDA). Spectralis (Heidelberg Engineering) obtained FDA clearance in September 2018. The majority of previous studies used the AngioVue system, which has a vessel density metric similar to that of the perfusion density in the AngioPlex system. Magrath, *et al*.^22^ concluded that results from different OCTA machines are not interchangeable. AngioPlex was used in the current study because it was observed to be superior to other OCTA systems with fewer artefacts^23–25^. Enders, *et al*.^26^ evaluated different artefacts of the AngioPlex system in healthy eyes and eyes with various retinopathies. Over 90% of the OCTA datasets were sufficient to excellent for interpretation of retinal microvasculature. Although motion and banding artefacts were relative common in the superficial capillary plexus, they could be identified easily from careful examination of *en face* slab images. Segmentation error was not common in the superficial retinal layer, and more common in eyes with pathologies. Nevertheless, from the current finding, it is still not advisable to take only one scan during OCTA measurement in healthy eyes.

OCTA quantitative measures may be presented using the Early Treatment of Diabetic Retinopathy Study (ETDRS) circular grid^11,12,27^, or square grid^28–30^, or circular annulus^19^. We propose that given the expanding research interest in OCTA it is advisable for an accepted standardized measurement region to be used. As the ETDRS circular grid is foveally centred and can be easily correlated with structural metrics such as retinal layer thicknesses, it may be the most prudent choice.

There are some limitations in the current study. A 3 × 3 mm ETDRS grid was used rather than a full 6 × 6 mm ETDRS grid, because the former pattern has a higher transverse resolution (12.2 µm and 17.1 µm respectively). Although AngioVue comes with a higher transverse resolution of 9.9 µm, it has more motion artefacts and longer execution time^23,24^. There are several OCTA machines on the market and only the Zeiss Cirrus 5000 with Angioplex model was evaluated. In addition, Sampson, *et al*.^31^ have previously reported that axial length can affect image magnification and therefore the OCTA measurements analysed. In our study, no correction was made to measurements based on axial length as OCTA findings are not compared in different refractive groups. Moreover, the 3 × 3 mm field is reported to be small enough to be relatively robust against changes in axial length. Finally, foveal avascular zone, another parameter provided by the AngioPlex, was not evaluated in our study. It has been previously reported that FAZ repeatability is not influenced by image size correction^17^.

In conclusion, while for several parameters a single measurement could provide a reasonable estimate of the true value, we would recommend averaging three OCTA measurements/acquisitions to reduce the amount of discrepancy between a single measurement and the ‘true value’ (in our case the mean of five measurements for the study of perfusion and vessel densities).

## Data Availability

The datasets used and analysed during the current study are available from the corresponding author on reasonable request.
